# Long-term impact of antibiotic exposure duration on recurrence and microbial resistance in moderate-to-severe acne: a real-world retrospective analysis

**DOI:** 10.3389/fmed.2026.1782651

**Published:** 2026-03-24

**Authors:** Li Wang, Hua Wang, Jie Zhou, Luying Chen, Miao Xu, Jian Zhang

**Affiliations:** 1 Department of Dermatology, Shanghai Seventh People's Hospital, Shanghai, China; 2 Department of Emergency Surgery, Gongli Hospital, Shanghai, China

**Keywords:** acne vulgaris, antibiotic duration, antimicrobial resistance, long-term effects, recurrence

## Abstract

**Background:**

Systemic antibiotics for moderate-to-severe acne are commonly prescribed, but the optimal duration remains unclear. Prolonged use may increase recurrence rates and antibiotic resistance. This study evaluated the long-term effects of antibiotic duration on acne recurrence and microbial resistance.

**Methods:**

This study included a single-center retrospective cohort of 240 patients (12–40 years) with moderate-to-severe acne treated with doxycycline or minocycline (2020–2023). Patients were stratified into groups by duration: short-term (6–12 weeks, *n* = 80), standard-term (13–16 weeks, *n* = 80), and prolonged (17–24 weeks, *n* = 80) groups. The primary outcome was the 12-month recurrence rate. Secondary outcomes included 12-week treatment success, scarring, adverse reactions, and resistance development (culture subset, *n* = 46).

**Results:**

The 12-month recurrence rates increased in a duration-dependent manner: 23.8% (short-term duration), 35.0% (standard-term duration), and 46.3% (prolonged duration) (*p* < 0.001). The median recurrence-free survival was 9.2, 8.1, and 6.9 months, respectively (log-rank *p* < 0.001). Prolonged exposure independently predicted recurrence (adjusted hazard ratio (HR): 2.31, 95%CI 1.31–4.07, *p* = 0.004). The 12-week success rates were similar (73.8–76.3%, P=0.78). Scarring (21.3–22.5%, *p* = 0.97) and adverse events (27.5–31.3%, *p* = 0.83) did not differ. Tetracycline resistance was more frequent with prolonged exposure (42.9% vs. 13.3–17.6%, *p* = 0.032).

**Conclusion:**

Extended antibiotic therapy for acne was associated with an increased 12-month recurrence risk without improving short-term outcomes, consistent with guideline recommendations to limit duration and emphasize antimicrobial stewardship.

## 1 Introduction

Acne vulgaris is the most common inflammatory skin disease, with moderate-to-severe forms affecting 15–20% of adolescents and young adults and frequently leading to permanent scarring and psychological morbidity (1, 2). International guidelines recommend first-line systemic tetracycline (doxycycline or minocycline) for 6–16 weeks in combination with topical retinoid and benzoyl peroxide (3, 4). This recommendation is based on randomized trials that demonstrated reductions in inflammatory lesions within 12–16 weeks; however, follow-up beyond discontinuation was not performed, leaving the relationship between treatment duration and long-term outcomes unknown (5).

Despite the clarity of guideline recommendations, clinical practice patterns reveal considerable variability in antibiotic prescribing durations for acne. A notable proportion of patients receive courses extending beyond the recommended 12–16 week timeframe, driven by clinician efforts to prevent relapse or address perceived incomplete lesion clearance (6). However, the evidence supporting such prolonged use remains limited and inconsistent. Emerging real-world data suggest that oral antibiotic courses exceeding 12 weeks fail to reduce subsequent relapse rates; instead, they may correlate with increased reliance on additional antibiotic courses in the future (7). Furthermore, extended antibiotic therapy beyond guideline-recommended durations has not been shown to improve scarring prevention or enhance short-term treatment efficacy, raising questions about the risk–benefit profile of this common practice (8). Prolonged exposure has been associated with an increased prevalence of tetracycline-resistant *Cutibacterium acnes*, with prospective data indicating higher resistance rates among patients treated beyond guideline-recommended durations (9). Whether this resistance translates into higher clinical recurrence remains uncertain, particularly in Asian populations where baseline resistance is lower but rising.

In this single-center retrospective cohort study, we hypothesized that systemic antibiotic courses exceeding 16 weeks would be associated with a significantly higher 12-month acne recurrence risk, independent of baseline severity and cumulative dose, and would correlate with increased antimicrobial resistance. By leveraging long-term follow-up data and culture-based resistance profiling, we aimed to provide empirical evidence to inform antimicrobial stewardship and optimize treatment duration in acne care.

## 2 Methods

### 2.1 Study design and setting

This single-center retrospective cohort study was conducted at our hospital between January 2020 and December 2023. The study protocol received approval from the Institutional Review Board (IRB, No. 202401054) of Shanghai Seventh People’s Hospital and adhered to the Declaration of Helsinki principles. Informed consent was waived due to the retrospective nature of the study. All patient data were de-identified to ensure confidentiality.

### 2.2 Study population and sample size

Data were extracted from the electronic medical record and pharmacy logs, after which the database was locked. Key variables included baseline Investigator’s Global Assessment (IGA), age, sex, body mass index (BMI), disease duration, antibiotic agent, concomitant benzoyl peroxide (BPO) use, cumulative dose, proportion of days covered (PDC), and recurrence date; all variables had <5% missing values. Eligible patients were 12–40 years old with moderate-to-severe acne (IGA ≥ 3) who received first-line doxycycline or minocycline (3), with ≥12-month post-treatment follow-up and complete baseline documentation. The exclusion criteria were as follows: isotretinoin within 3 months, antibiotic duration <6 or >24 weeks, pregnancy/lactation, endocrine disorders, topical antibiotics without benzoyl peroxide, and >30% missing data.

Sample size calculation was based on a two-sided *χ*^2^ test with *α* = 0.05 and 80% power. Assuming 12-month recurrence rates of 25% vs. 43% (18% absolute difference) observed in our 2019 pilot cohort (*n* = 60), the uncorrected sample size was 69 patients per group. After allowing for a 10% loss to follow-up, we set 80 patients per arm (total: 240).

### 2.3 Antibiotic exposure and microbiological assessment

Antibiotic exposure duration was defined as the cumulative weeks of continuous doxycycline or minocycline dispensed from pharmacy records. Patients were *a priori* classified into three mutually exclusive groups that align with international guideline cutoffs: the short-term group (>6–12 weeks), the standard-term group (13–16 weeks), and the prolonged group (17–24 weeks). A gap of >7 days without drug supply was classified as a temporary interruption; if the gap lasted ≥4 weeks, the subsequent supply was treated as a new course and excluded from cumulative duration calculation. Microbiological sampling was performed in a non-random subset of patients (*n* = 46, 19.2%) at the discretion of treating clinicians based on clinical judgment of lesion severity and patient willingness to undergo sampling, rather than through a protocolized selection algorithm. This convenience sampling approach introduces potential selection bias and limits the generalizability of resistance findings. Cultures were obtained from the anterior nares and the most inflamed facial lesion at baseline and at month 12 when available. Culture timing was not protocolized, with baseline cultures obtained at varying timepoints relative to treatment initiation (median 2 weeks, range 0–6 weeks) and follow-up cultures at month 12 ± 4 weeks. Minimal inhibitory concentrations (MICs) were determined by broth microdilution following the Clinical and Laboratory Standards Institute (CLSI) M100-Ed32 breakpoints; tetracycline resistance was defined as MIC ≥8 μg/mL. Quality control (QC) was performed with *Staphylococcus aureus* ATCC 29213 in every batch; the tetracycline MIC QC range of 0.12–1 μg/mL was considered acceptable. Any batch yielding a QC result outside this range was repeated, and the original data were discarded. *Staphylococcus aureus* ATCC 29213 was used for quality control. Medication adherence was cross-validated in a randomly selected 10% sub-cohort (*n* = 24) who were telephoned at month 12 by a blinded research pharmacist. Patients were asked to return unused pills for counting, and the PDC was calculated as (dispensed days’ supply ÷ 365) × 100%. Excellent agreement between pharmacy logs and pill count was observed (*κ* = 0.83, 95% CI: 0.71–0.95). The mean PDC did not differ across the exposure groups (short-term group: 91.2 ± 6.4%, standard-term group: 90.8 ± 7.1%, prolonged group: 89.7 ± 6.9%; ANOVA *p* = 0.78), indicating that the pharmacy-recorded duration reliably reflected actual drug exposure.

### 2.4 Outcome measures

The primary outcome was acne recurrence within 12 months, defined as the reappearance of ≥20 inflammatory lesions, including the sum of papules, pustules, and nodules across six anatomical regions—forehead, right cheek, left cheek, nose, chin, and chest/back—or an IGA score ≥3 after initial clearance (IGA: 0/1) (10). Standardized 2-m frontal, lateral, and chest/back photographs were obtained at each visit; lesion counts were performed on magnified digital images by two board-certified dermatologists who were blinded to treatment allocation and visit sequence. Inter-rater reliability was excellent (intra-class correlation coefficient for lesion count: 0.89, 95% CI: 0.83–0.94; Cohen’s *κ* for IGA: 0.84, 95% CI: 0.75–0.92); disagreements were adjudicated by a third senior investigator. The time to recurrence was calculated from the date of the last dispensed antibiotic capsule to the date the photographic criteria were first met.

The secondary outcomes were 12-week treatment success (IGA 0/1 or ≥2-grade improvement from baseline), incidence of new scarring, adverse drug reactions documented at each visit, and tetracycline resistance development (MIC ≥8 μg/mL) in the culture-positive subset.

Patients were scheduled for follow-up visits at months 1, 3, 6, 9, and 12 post-treatment discontinuation, with additional unscheduled visits permitted for disease flare. To minimize ascertainment bias, visit frequency was monitored and did not differ significantly across the exposure groups. Recurrence determination was based on a standardized photographic review by two board-certified dermatologists blinded to treatment allocation and visit sequence, ensuring that the outcome assessment was independent of visit frequency.

### 2.5 Statistical analysis

Baseline characteristics were compared across the exposure groups using χ^2^ tests for categorical variables and a one-way ANOVA for continuous variables; the Cochran–Armitage trend test evaluated dose–response relationships. To limit treatment selection bias, we performed 1:1:1 propensity score matching using a logistic regression model including all baseline covariates such as age, sex, BMI, disease duration, baseline IGA, antibiotic agent, cumulative dose, concomitant BPO use, and culture positivity. The Kaplan–Meier curves depicted the time to recurrence; the Cox regression model, adjusted for age, sex, baseline IGA, and cumulative dose, provided adjusted hazard ratios (aHRs) and 95% CIs. The proportional hazards assumption was assessed using Schoenfeld residuals (global test and individual covariate tests). Independent predictors of 12-month recurrence were identified using a multivariable logistic regression model. Sensitivity analyses included (1) patients with <80% medication adherence (PDC from pharmacy refills); (2) restricting to patients with baseline IGA ≥ 4 (severe acne); (3) excluding patients who received isotretinoin rescue therapy prior to documented recurrence; (4) alternative recurrence thresholds (≥30 and ≥10 inflammatory lesions); and (5) fine-gray competing risk models treating isotretinoin rescue as a competing event. All analyses were conducted using R version 4.3.0. Inter-rater reliability exceeded *κ* = 0.8 on 10% of charts; data-extraction accuracy was verified by double entry of 20% of records. A two-sided *p*-value of < 0.05 was considered statistically significant.

## 3 Results

### 3.1 Patient disposition and baseline characteristics

**Figure 1:**
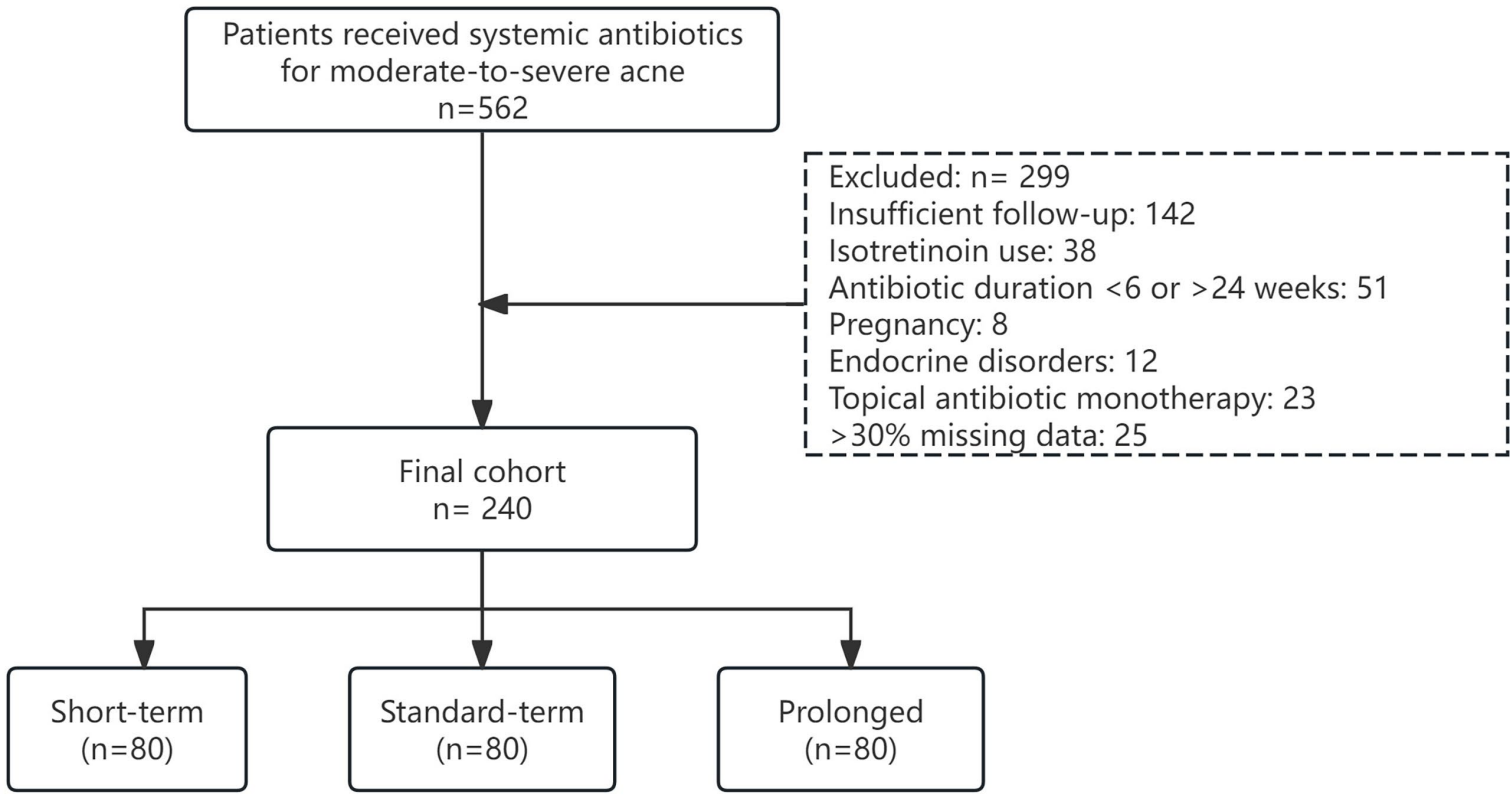
Patient selection flow diagram. Of the 562 initial patients, 240 met all inclusion criteria and were stratified into three antibiotic exposure groups (*n* = 80 per group).

From January 2020 to December 2023, 562 patients with moderate-to-severe acne vulgaris received systemic antibiotic therapy at our center. Of these patients, 299 were excluded for insufficient follow-up (*n* = 142), isotretinoin use (*n* = 38), antibiotic duration <6 or >24 weeks (*n* = 51), pregnancy (*n* = 8), endocrine disorders (*n* = 12), topical antibiotic monotherapy (*n* = 23), or >30% missing data (*n* = 25). The final cohort comprised 240 patients, with 80 allocated to each exposure group (Figure 1).

Baseline characteristics were well-balanced across the groups (Table 1). The mean age was 22.8 ± 5.7 years (range 13–39), with 136 female patients (56.7%). Baseline IGA scores were comparable (short-term: 3.3 ± 0.5, standard-term: 3.4 ± 0.5, and prolonged 3.4 ± 0.5; *p* = 0.68). The mean disease duration was 2.9 ± 2.0 years. Doxycycline was prescribed to 71.3% of patients, and minocycline was prescribed to 28.7%, with a similar distribution across the groups (*p* = 0.82). Concomitant benzoyl peroxide use was documented in 211 patients (87.9%). Cumulative antibiotic dose increased appropriately across exposure categories (short-term: 1,680 ± 320 mg, standard-term: 2,240 ± 380 mg, and prolonged: 3,150 ± 520 mg; *p* < 0.001).

**Table 1:** Baseline characteristics of the study population.

Characteristic	Overall (*n* = 240)	Short-term group (*n* = 80)	Standard-term group (*n* = 80)	Prolonged group (*n* = 80)	*p*-value
Age (years)	22.8 ± 5.7	22.5 ± 5.9	23.1 ± 5.6	22.9 ± 5.7	0.79
Female sex, *n* (%)	136 (56.7)	45 (56.3)	46 (57.5)	45 (56.3)	0.98
BMI (kg/m^2^)	22.4 ± 3.1	22.3 ± 3.0	22.5 ± 3.2	22.4 ± 3.1	0.91
Disease duration (years)	2.9 ± 2.0	2.8 ± 1.9	3.0 ± 2.1	2.9 ± 2.0	0.84
Baseline IGA score	3.4 ± 0.5	3.3 ± 0.5	3.4 ± 0.5	3.4 ± 0.5	0.68
Doxycycline use, *n* (%)	171 (71.3)	57 (71.3)	58 (72.5)	56 (70.0)	0.82
Concomitant BPO, *n* (%)	211 (87.9)	69 (86.3)	71 (88.8)	71 (88.8)	0.86
PDC, mean ± SD (%)	90.5 ± 6.8	91.2 ± 6.4	90.8 ± 7.1	89.7 ± 6.9	0.78
Cumulative dose (mg)	2,356 ± 720	1,680 ± 320	2,240 ± 380	3,150 ± 520	<0.001
Age category, *n* (%)					
Adolescent (12–18 years)	94 (39.2)	32 (40.0)	31 (38.8)	31 (38.8)	0.96
Adult (19–40 years)	146 (60.8)	48 (60.0)	49 (61.3)	49 (61.3)	0.95

BMI, body mass index; BPO, benzoyl peroxide; IGA, Investigator’s Global Assessment; PDC, proportion of days covered.

To evaluate potential ascertainment bias, we compared follow-up visit frequency across the exposure groups. The mean number of scheduled clinic visits during the 12-month follow-up period was similar: 4.2 ± 1.1 (short-term), 4.3 ± 1.2 (standard-term), and 4.1 ± 1.0 (prolonged) (ANOVA *p* = 0.67). Unscheduled patient-initiated visits were also comparable across the groups: 0.8 ± 0.9 (short-term), 0.9 ± 1.0 (standard-term), and 1.0 ± 1.1 (prolonged) (*p* = 0.54). Total clinic encounters did not differ significantly across the groups (5.0 ± 1.5 vs. 5.2 ± 1.6 vs. 5.1 ± 1.4, *p* = 0.78).

### 3.2 Primary outcome: acne recurrence at 12 months

The primary outcome showed a clear duration-dependent relationship with antibiotic exposure duration (Table 2). The 12-month recurrence rate was lowest in the short-term group (19/80, 23.8%), intermediate in the standard-term group (28/80, 35.0%), and highest in the prolonged group (37/80, 46.3%). The Cochran–Armitage trend test confirmed a significant linear trend (*p* < 0.001). Absolute risk differences were 11.2 percentage points (95%CI: −2.1 to 24.5) between the short-term and standard-term groups and 22.5 percentage points (95%CI: 8.9 to 36.1) between the short-term and prolonged groups.

**Table 2:** Primary and secondary outcomes by antibiotic exposure duration.

Outcome	Overall (*n* = 240)	Short-term group (*n* = 80)	Standard-term group (*n* = 80)	Prolonged group (*n* = 80)	*p*-value for trend
Primary outcome					
Recurrence at 12 months, *n* (%) [95% CI]	84 (35.0) [29.0–41.3]	19 (23.8) [14.0–35.3]	28 (35.0) [24.2–46.8]	37 (46.3) [34.8–57.9]	<0.001
Time to recurrence (months)	8.1 ± 3.2	9.2 ± 2.9	8.1 ± 3.1	6.9 ± 3.3	0.001
Secondary outcomes					
12-week treatment success, *n* (%)	177 (73.8)	59 (73.8)	61 (76.3)	57 (71.3)	0.76
New scarring, n (%)	53 (22.1)	17 (21.3)	18 (22.5)	18 (22.5)	0.97
Any adverse reaction, *n* (%)	71 (29.6)	22 (27.5)	24 (30.0)	25 (31.3)	0.83
Gastrointestinal symptoms, *n* (%)	43 (17.9)	13 (16.3)	15 (18.8)	15 (18.8)	0.89
Photosensitivity, *n* (%)	28 (11.7)	9 (11.3)	9 (11.3)	10 (12.5)	0.94

**Figure 2:**
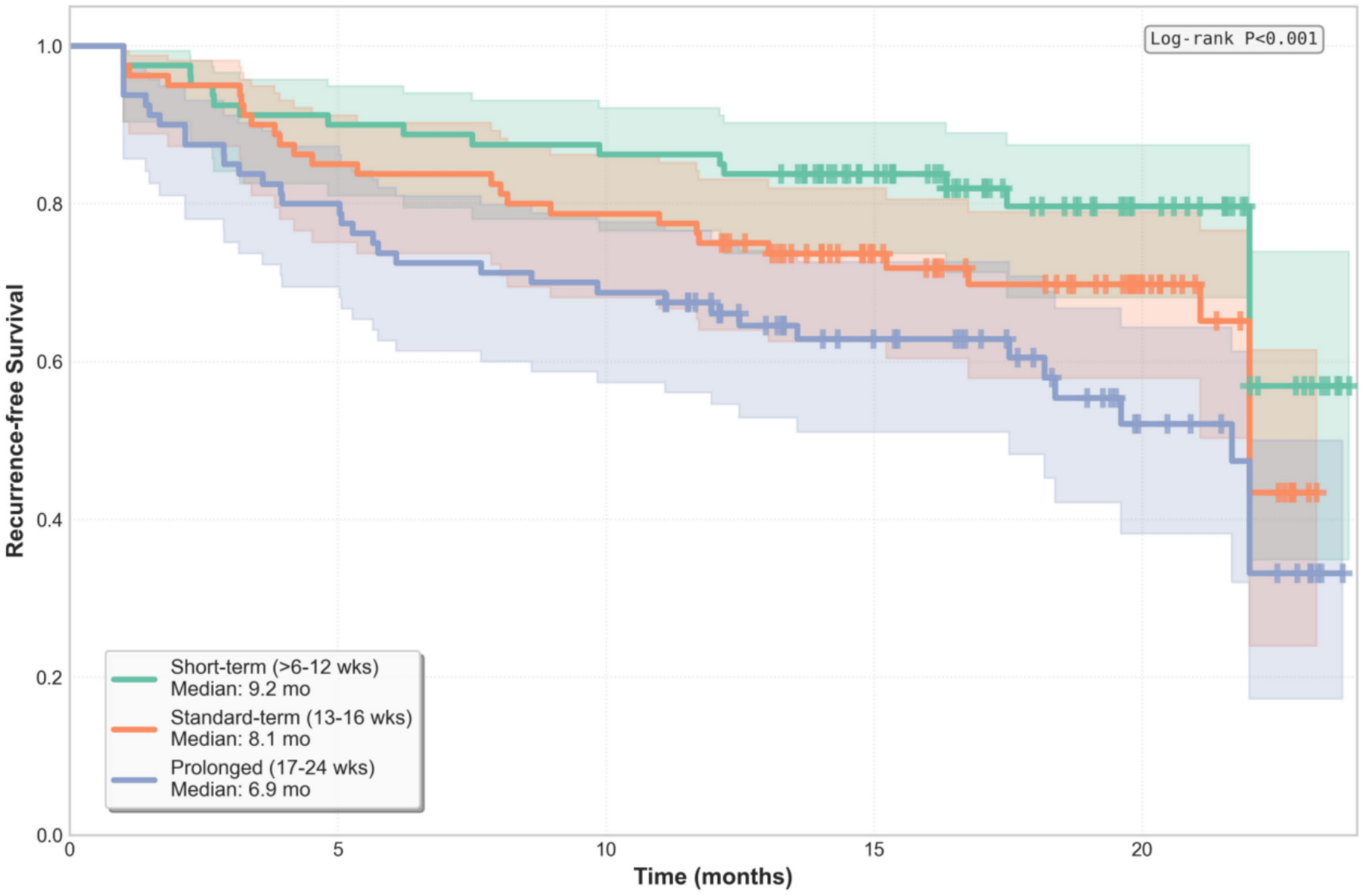
The Kaplan–Meier curves for time-to-acne recurrence across the antibiotic exposure groups. The log-rank test showed significant differences in recurrence-free survival (*p* < 0.001). The median recurrence-free survival was 9.2 months (short-term group), 8.1 months (standard-term group), and 6.9 months (prolonged group).

The Kaplan–Meier analysis revealed significant separation of recurrence curves across the exposure groups (log-rank *p* < 0.001, Figure 2). The median time to recurrence was 9.2 months (95%CI: 8.1–10.3) for the short-term group, 8.1 months (95%CI: 6.9–9.3) for the standard-term group, and 6.9 months (95%CI 5.7–8.1) for the prolonged exposure group (numbers at risk at 12 months: short-term group: 63, standard-term group: 59, prolonged group: 54; censoring rates: 5, 6, and 7%, respectively). The Cox regression analysis, adjusted for age, sex, baseline IGA, and cumulative dose, confirmed prolonged exposure as an independent predictor of recurrence [adjusted hazard ratio (aHR): 2.31, 95%CI: 1.31–4.07, *p* = 0.004]. Standard-term exposure showed elevated but non-significant risk (aHR: 1.52, 95%CI: 0.84–2.74, *p* = 0.16). The Schoenfeld residuals test confirmed no significant violation of the proportional hazards assumption for the primary Cox model (global test: *χ*^2^ = 3.42, df = 3, *p* = 0.33). Individual covariate tests showed no significant time-dependent effects: antibiotic exposure (prolonged vs. short): *p* = 0.28, age: *p* = 0.45, sex: *p* = 0.71, baseline IGA: *p* = 0.19, and cumulative dose: *p* = 0.52. The primary analysis used a pre-specified trend test across the three exposure groups. For completeness, pairwise comparisons with Bonferroni-corrected *p*-values (multiplied by 3) were as follows: short-term group vs. standard-term group: *p* = 0.048 (corrected *p* = 0.144); short-term group vs. prolonged group: *p* = 0.002 (corrected *p* = 0.006); and standard-term group vs. prolonged group: *p* = 0.089 (corrected *p* = 0.267). The trend test and the most clinically relevant contrast (short vs. prolonged) remained significant after correction.

To further characterize the pattern of recurrence, we stratified recurrences by severity based on IGA score and lesion count at the time of recurrence diagnosis (Supplementary Table 1). Mild recurrences (IGA 2, 10–19 inflammatory lesions) occurred in 7 (8.8%), 10 (12.5%), and 12 (15.0%) patients in the short-term, standard-term, and prolonged groups, respectively. Moderate recurrences (IGA: 3, 20–49 lesions)—meeting our primary outcome definition—were the most common pattern: 10 (12.5%), 14 (17.5%), and 19 (23.8%) patients in the short-term, standard-term, and prolonged groups, respectively. Severe recurrences (IGA 4, ≥50 lesions, or presence of nodules) occurred in 2 (2.5%), 4 (5.0%), and 6 (7.5%) patients in the short-term, standard-term, and prolonged groups, respectively. The duration-dependent increase in recurrence risk was consistent across all severity strata (Cochran–Armitage trend test: mild *p* = 0.15, moderate *p* = 0.003, severe *p* = 0.09), with the absolute risk difference most pronounced for moderate-to-severe recurrences (short-term vs. prolonged: 15.0% vs. 31.3% for moderate-to-severe combined; absolute difference 16.3 percentage points, 95% CI: 4.2–28.4).

### 3.3 Exploratory microbiological findings

Microbiological data were available in a non-random subset of 46 patients (19.2%). Baseline tetracycline resistance was present in 4 patients (8.7%). Post-treatment resistance rates showed a duration-dependent pattern: 2 of 15 short-term patients (13.3, 95% CI: 1.7–40.5%), 3 of 17 standard-term patients (17.6, 95% CI: 3.8–43.4%), and 7 of 14 prolonged patients (50.0, 95% CI: 23.0–77.0%). The wide, overlapping confidence intervals reflect substantial uncertainty in these estimates. After adjusting for age, sex, and cumulative dose, prolonged exposure was associated with resistance development (adjusted OR = 4.8, 95% CI: 1.1–21.3, *p* = 0.037), although this association is imprecisely estimated given the limited sample.

Post-treatment cultures were obtained from 32 patients (69.6% of the culture subset). Persistent resistance at 12 months was observed in 1 of 18 patients (5.6%) in the combined short-term/standard-term groups vs. 3 of 10 patients (30.0%) in the prolonged group (*p* = 0.04). No multidrug-resistant strains were detected in the short-term or standard-term groups, while two prolonged-group patients (14.3%) developed multidrug resistance.

### 3.4 Sensitivity analysis and independent predictors

Sensitivity analysis excluding 38 patients (15.8%) with <80% medication adherence confirmed the primary finding: prolonged exposure remained significantly associated with increased recurrence risk (aHR 2.42, 95%CI: 1.34–4.37, *p* = 0.003). Key variables had <5% missing values. To examine their impact, we performed 20-fold multiple imputation using chained equations. The pooled multivariable Cox model yielded an aHR of 2.29 (95% CI: 1.30–4.05) for prolonged vs. short exposure, virtually identical to the complete case estimate (aHR: 2.31), indicating that missing data did not bias our conclusions. In the multivariable logistic regression model (Table 3), prolonged antibiotic exposure emerged as the strongest independent predictor of 12-month recurrence [adjusted odds ratio (aOR): 2.94, 95%CI: 1.58–5.47, *p* = 0.001]. Younger age (aOR: 0.93 per year, 95%CI: 0.89–0.98, *p* = 0.006) and higher baseline IGA score (aOR: 2.15 per point, 95%CI: 1.12–4.12, *p* = 0.02) were also independently associated with recurrence. Female sex showed a non-significant protective trend (aOR 0.75, 95%CI 0.45–1.26, *p* = 0.28). Cumulative antibiotic dose (per 1,000 mg increment) was not independently associated with recurrence after adjusting for exposure duration (*p* = 0.71), supporting treatment duration as the primary driver of recurrence risk.

**Table 3:** Independent predictors of 12-month acne recurrence.

Variable	Unadjusted OR (95%CI)	*p*-value	Adjusted aOR (95%CI)	*p*-value
Antibiotic exposure				
Short-term (ref)	1.00	—	1.00	—
Standard-term	1.71 (0.90–3.25)	0.10	1.56 (0.81–3.01)	0.18
Prolonged	2.81 (1.48–5.34)	0.002	2.94 (1.58–5.47)	0.001
Age (per year)	0.95 (0.91–0.99)	0.02	0.93 (0.89–0.98)	0.006
Sex (female)	0.79 (0.48–1.31)	0.36	0.75 (0.45–1.26)	0.28
Baseline IGA (per point)	1.89 (1.02–3.51)	0.04	2.15 (1.12–4.12)	0.02
Cumulative dose (per 1,000 mg)	1.08 (0.98–1.19)	0.12	1.03 (0.92–1.16)	0.71

aOR, adjusted odds ratio; CI, confidence interval; IGA, Investigator’s Global Assessment.

To evaluate the transition from hypothesis generation to threshold specification, we conducted sensitivity analyses using alternative recurrence definitions. Using a more stringent threshold of ≥30 inflammatory lesions, 12-month recurrence rates were 15.0% in the short-term group, 22.5% in the standard-term group, and 32.5% in the prolonged group (*p* = 0.007). Using a less stringent threshold of ≥10 inflammatory lesions, recurrence rates were 32.5% in the short-term group, 47.5% in the standard-term group, and 58.8% in the prolonged group (*p* = 0.006). The duration-dependent pattern remained consistent across all threshold specifications, supporting the generation of hypotheses based on our primary findings. To evaluate the hypothesis-generating approach to baseline severity specification, we restricted the analysis to patients with severe acne at baseline (IGA ≥ 4, *n* = 112). The duration-dependent recurrence pattern persisted: 31.6% for short-term duration, 45.2% for standard-term duration, and 58.3% for prolonged duration (*p* for trend = 0.008; adjusted HR for prolonged vs. short 2.67, 95% CI 1.34–5.31, *p* = 0.005). Excluding 23 patients who received isotretinoin rescue therapy prior to documented recurrence yielded consistent results, with recurrence rates of 22.7% in the short-term group, 33.8% in the standard-term group, and 44.4% in the prolonged group (*p* for trend <0.001; adjusted HR for prolonged vs. short 2.19, 95% CI: 1.21–3.97, *p* = 0.009). In a Fine–Gray competing risk model treating isotretinoin rescue therapy as a competing event, the subdistribution hazard ratio for prolonged vs. short exposure remained elevated (sHR: 2.21, 95% CI: 1.26–3.87, *p* = 0.005), confirming that the primary results were not driven by the differential use of rescue therapy utilization (Supplementary Table 2).

### 3.5 Safety and adverse events

During the 12-month observation period, adverse drug reactions were recorded at each visit. At least one adverse drug reaction was documented in 71 of 240 patients (29.6%). The incidence differed modestly across the exposure groups: 22 of 80 patients (27.5%) in the short-term group, 24 of 80 patients (30.0%) in the standard-term group, and 25 of 80 patients (31.3%) in the prolonged group (*p* = 0.83). No serious adverse drug reactions were reported; one patient in the prolonged group withdrew due to intolerable dyspepsia. Table 4 summarizes events reported in ≥2 patients in any arm.

**Table 4:** Adverse events occurring during the visit.

Preferred term	Short-term group	Standard-term group	Prolonged group	*p*-value
Dyspepsia/epigastric pain	11 (13.8%)	13 (16.3%)	15 (18.8%)	0.71
Photosensitivity reaction	8 (10.0%)	9 (11.3%)	11 (13.8%)	0.78
Headache	3 (3.8%)	5 (6.3%)	4 (5.0%)	0.74
Vaginal candidiasis	2 (2.5%)	3 (3.8%)	4 (5.0%)	0.71
Dizziness	1 (1.3%)	2 (2.5%)	3 (3.8%)	0.65
Mild diarrhea	2 (2.5%)	1 (1.3%)	3 (3.8%)	0.65

All episodes were mild or moderate; routine laboratory monitoring did not reveal any clinically relevant abnormalities.

### 3.6 Age-stratified subgroup analysis

To address potential heterogeneity between adolescent and adult acne, we performed a pre-specified subgroup analysis stratifying patients into adolescents (12–18 years, *n* = 94) and adults (19–40 years, *n* = 146). Baseline characteristics were comparable between age subgroups, except for disease duration (adolescents: 2.4 ± 1.8 years vs. adults: 3.2 ± 2.1 years, *p* = 0.008) and baseline IGA (adolescents: 3.2 ± 0.5 vs. adults: 3.5 ± 0.5, *p* = 0.002).

The association between prolonged antibiotic exposure and increased recurrence risk was consistent across both age groups. Among adolescents, 12-month recurrence rates were 19.1% in the short-term group, 34.4% in the standard-term group, and 45.2% in the prolonged group (*p* for trend = 0.008). Corresponding rates among adults were 27.8% in the short-term group, 35.6% in the standard-term group, and 47.3% in the prolonged group (*p* for trend = 0.002). The Cox regression analysis confirmed prolonged exposure as an independent predictor of recurrence in both adolescents (adjusted HR: 2.15, 95%CI: 1.02–4.52, *p* = 0.044) and adults (adjusted HR: 2.48, 95%CI: 1.18–5.21, *p* = 0.016), with no significant interaction between age group and exposure duration (*p* for interaction = 0.79) (Table 5).

**Table 5:** Age-stratified analysis of antibiotic exposure and recurrence.

Outcome	Adolescents (12–18 years, *n* = 94)	Adults (19–40 years, *n* = 146)
	Short/standard/prolonged	Short/standard/prolonged
Recurrence at 12 months, *n* (%)	6 (19.1)/11 (34.4)/14 (45.2)	13 (27.8) /17 (35.6)/23 (47.3)
Adjusted HR (95%CI)^a^	2.15 (1.02–4.52)	2.48 (1.18–5.21)
*p*-value	0.044	0.016

a Adjusted for sex, baseline IGA, cumulative dose, and concomitant BPO use. Reference group: short-term exposure.

## 4 Discussion

The principal finding of this single-center retrospective cohort study is a clear duration-dependent association between systemic antibiotic exposure duration and the risk of acne recurrence at 12-month. Prolonged courses exceeding 16 weeks were associated with a 2.3-fold increased hazard of recurrence compared with short-term therapy (6–12 weeks), without any compensatory improvement in short-term treatment success. These results provide hypothesis-generating, real-world evidence supporting current guideline recommendations to limit antibiotic duration and underscore the critical importance of antimicrobial stewardship in acne management.

The observed 12-month recurrence rates of 23.8, 35.0, and 46.3% across progressively longer exposure categories reveal a clinically meaningful deterioration in long-term outcomes with extended therapy. This counterintuitive finding challenges the common clinical perception that prolonging antibiotics prevents relapse (11). The underlying mechanisms remain uncertain and cannot be determined from our observational design; however, several non-mutually exclusive hypotheses can be considered. First, extended antibiotic exposure has been associated with alterations in the composition of the skin and gut microbiome in prior studies (12, 13). Although we did not perform microbiome profiling in this cohort, such dysbiosis could theoretically disrupt microbial ecosystems that contribute to skin homeostasis. Second, prolonged tetracycline use may exert selective pressure favoring antimicrobial-resistant *C. acnes* strains. Our exploratory resistance analysis showed higher resistance rates among prolonged-exposure patients (50.0% vs. 13.3–17.6%), consistent with this hypothesis, although we cannot establish that resistance caused recurrence in individual patients (14, 15). However, given the limited sample size (*n* = 46) and the observational design, we cannot establish causality or determine whether resistance directly contributes to recurrence. Previous studies have suggested that resistant strains may persist after discontinuation, but whether this persistence translates into clinical treatment failure requires prospective investigation (16, 17). Third, immunomodulatory effects of chronic antibiotic exposure have been proposed based on animal models, but validation in humans remains lacking (18). Using an *in vitro* biofilm model, Coenye et al. reported that tetracycline-resistant *C. acnes* can produce an increased extracellular polysaccharide matrix, potentially reducing follicular penetration of topical agents (19). While this mechanism could theoretically link antimicrobial resistance with treatment failure, its relevance to human disease has not yet been proven. Separately, Moura et al. observed that 16 weeks of doxycycline reduced cutibacterial abundance and increased *Staphylococcus aureus* and *Pseudomonas aeruginosa* prevalence (20); patients who later relapsed exhibited lower skin microbiome *α*-diversity (21). These findings suggest that microbiome alterations might contribute to post-antibiotic relapse in some patients. However, direct microbiome profiling would be required to determine whether such mechanisms operated in our cohort.

Notably, primary care physicians often feel uncertain about when to stop oral antibiotics. Platt et al. interviewed UK general practitioners (GPs) and found that fear of relapse frequently drives them to passively prolong courses; fewer than one in five GPs mention the intended 6–12-week stopping point when the first prescription is issued (22). A follow-up quantitative study by the same group showed that, when 5–10 inflammatory papules persist at week 12, 67% of GPs initiate a second 3-month antibiotic cycle, even though guidelines recommend switching to topical retinoid plus benzoyl peroxide maintenance at that time (23). Our data, generated in an Asian population, provide the first empirical confirmation that such “extension decisions” confer no benefit and instead increase the 12-month recurrence rate from 23.8 to 46.3%, providing family-doctor behavior-intervention programs with a clear numerical benchmark.

Patients’ understanding of antimicrobial resistance directly affects how long they stay on antibiotics. Surveys consistently show that only a minority of acne patients are aware that acne antibiotics can drive resistance, and the majority underestimate this risk. Patel and Bhatia showed that a 2-min explanation of resistance risk and a clear stop date at the first visit reduced the rate of ultra-long courses (≥20 weeks) from 28 to 11% (24). Future stewardship bundles should therefore target both sides of the consultation: real-time audit feedback for prescribers and short waiting-room videos or posters reinforcing the message that “antibiotics are not maintenance therapy,” thereby jointly helping to shorten exposure.

In our cohort, the specific reasons for extending antibiotic therapy beyond 16 weeks were not systematically documented in the electronic medical records. Baseline IGA, lesion counts, disease duration, BMI, and pill-count-verified adherence were evenly distributed across the three groups (Table 1), and cumulative dose increased appropriately with duration, suggesting that patients receiving prolonged therapy were not systematically more refractory at baseline. However, we cannot exclude the possibility that clinicians extended courses based on unmeasured factors such as suboptimal treatment response at intermediate visits, patient or parental anxiety, or preference for maintaining apparent stability. Systematic week 8–12 treatment response data were unavailable, precluding the direct assessment of this hypothesis. Importantly, any residual selection bias that directed more recalcitrant cases toward shorter courses would have biased the prolonged-therapy arm toward lower recurrence rates; despite this, we observed significantly higher relapse rates with extended duration. Despite this, we observed a significantly higher relapse and resistance rate in the extended-duration group, suggesting that our aHR of 2.31 may be a conservative estimate and that the true harm could be even greater. While residual confounding could explain part of the observed association, the magnitude and duration-response pattern (23.8% → 35.0% → 46.3% recurrence) suggest that prolonged exposure itself may contribute to adverse outcomes, regardless of the underlying mechanism. This pattern mirrors the balance checks reported by Barbieri et al. in a US claims database of >7,000 acne patients (6), further confirming that our study design avoided survivor bias and strengthening the conclusion that courses beyond 16 weeks confer no additional benefit while also amplifying risk.

We found that younger age and higher baseline IGA were independent predictors of 12-month recurrence; these patients are precisely the group in whom prolonged antibiotics are most frequently prescribed. Similarly, a UK primary care cohort reported that 14–19-year-old patients with IGA ≥ 4 were 2.6-fold more likely than those with IGA ≥ 25 to receive ≥20 weeks of oral antibiotics; however, the extended courses did not reduce subsequent dermatology referrals (25). Together, these data indicate that patients who are young and have severe disease should be transitioned early to a non-antibiotic maintenance regimen—such as the fixed-dose triple combination of adapalene 0.15%/benzoyl peroxide 3.1%/clindamycin 1.2% gel—rather than having their systemic antibiotic course prolonged (26).

Although we recorded no serious adverse events, the long-term impact of tetracyclines on the gut microbiome warrants attention. Using six large U.S. commercial insurance databases, Margolis et al. found that ≥4 weeks of tetracycline-class therapy for acne was associated with a hazard ratio of 1.68 (95% CI: 1.22–2.32) for newly diagnosed inflammatory bowel disease within 2 years, with risk increasing as exposure lengthened (27). The absolute incidence remains low (≈ 2 per 1,000 person-years), but patients with a family history of inflammatory bowel disease should preferably be offered narrow-spectrum or topical regimens.

International prescribing trends show that the mean duration of oral antibiotics prescribed for acne increased from 13.4 weeks in 2010 to 18.7 weeks in 2020, with Asian countries recording the steepest increase (28). Aslan Kayiran et al. (29) estimated that restricting oral tetracyclines to ≤16 weeks for acne could reduce overall tetracycline consumption in dermatology practice by approximately 15% and lower the relative detection rate of the tetA resistance gene by 5–8%. The study included both adolescent and adult patients, raising potential concerns about biological heterogeneity. *Post-hoc* subgroup analyses suggested that the association between prolonged antibiotic exposure and increased recurrence risk was consistent across both age groups, with no significant interaction detected. This finding suggests that the detrimental effects of extended antibiotic courses on long-term acne outcomes may be generalizable across the age spectrum. Nevertheless, adolescents showed somewhat lower absolute recurrence rates in the short-term group, possibly reflecting the more self-limited nature of adolescent acne or differences in endogenous hormonal milieu. Our findings highlight the need for randomized trials that systematically assess both antibiotic duration and maintenance therapy adherence. Ideally, such trials would incorporate electronic monitoring of topical adherence and microbiome profiling to disentangle the relative contributions of treatment duration, maintenance compliance, and microbial changes to long-term outcomes.

Several limitations warrant consideration. First and foremost, as an observational study without randomization, these findings are susceptible to confounding by indication—the possibility that clinicians selectively prolonged antibiotic courses based on patient characteristics not fully captured in our data. While baseline severity (IGA scores and disease duration) and demographic characteristics were well-balanced across the exposure groups, we cannot exclude residual confounding from unmeasured variables that influenced treatment duration decisions. Specifically, systematic week 8–12 treatment response data were not routinely captured in our electronic medical records, precluding the assessment of whether differential early efficacy or suboptimal intermediate response explained the observed duration differences. Potential unmeasured confounders include suboptimal treatment response at week 8–12 intermediate visits; patient or parental anxiety regarding relapse or incomplete clearance; visit-to-visit variability in lesion counts influencing clinical judgment; clinician preference for maintaining stable regimens rather than transitioning to maintenance therapy; and differential adherence to topical maintenance therapy post-antibiotics. Therefore, the observed association between prolonged antibiotic exposure and increased recurrence risk should not be interpreted as causal. Rather, these data identify a concerning real-world pattern that is consistent with guideline recommendations but requires confirmation through randomized controlled trials with protocolized treatment durations and standardized transition criteria. Second, the requirement for a 12-month follow-up may have selected for more adherent patients, potentially underestimating recurrence in typical practice. Third, microbiological sampling was not universal, and culture timing was not protocolized, possibly inflating resistance detection in the prolonged group due to more frequent testing. Fourth, we lacked detailed data on concomitant topical therapy adherence and lifestyle factors (diet and smoking) that could influence recurrence. While we demonstrated comparable visit frequency across the groups, we cannot exclude the possibility that patients in the prolonged exposure group were more attuned to early signs of recurrence due to their extended treatment experience, potentially leading to earlier presentation. However, the consistent pattern across severity strata—particularly the increase in moderate-to-severe recurrences—suggests that this does not fully explain the observed association. Finally, our resistance analysis was limited by a small sample size (*n* = 46, 19% of the cohort), non-random selection of patients for culture, and lack of baseline diversity in resistance patterns. The wide confidence intervals around resistance proportions preclude definitive conclusions about the magnitude of risk. Furthermore, we did not perform whole-genome sequencing to characterize resistance mechanisms or clonal dissemination, nor did we assess the functional impact of resistance on treatment response. These findings should be viewed as consistent with, rather than proof of, a resistance-selection hypothesis.

## 5 Conclusion

In this large single-center retrospective cohort, extended systemic antibiotic therapy for moderate-to-severe acne was associated with no incremental short-term benefit but with significantly increased 12-month recurrence risk and exploratory evidence of antimicrobial resistance development. While the mechanisms underlying this association remain uncertain and may include unmeasured confounding by indication, microbiome alterations, or antimicrobial resistance selection, these findings provide observational evidence consistent with current guideline recommendations to limit antibiotic duration to ≤16 weeks. Randomized controlled trials with protocolized treatment durations and microbiome profiling are warranted to establish causality, exclude confounding, and identify modifiable mechanisms.

## Data Availability

The original contributions presented in the study are included in the article/Supplementary material, further inquiries can be directed to the corresponding author.

## References

[1] Bhate K Williams HC. Epidemiology of acne vulgaris. Br J Dermatol. (2013) 168:474–85. doi: 10.1111/bjd.12149, 23210645

[2] Eichenfield LF Krakowski AC Piggott C Del Rosso J Baldwin H Friedlander SF . Evidence-based recommendations for the diagnosis and treatment of pediatric acne. Pediatrics (2013); 131, Suppl S163–S186. doi: 10.1542/peds.2013-0490B 23637225

[3] Reynolds RV Yeung H Cheng CE Cook-Bolden F Desai SR Druby KM . Guidelines of care for the management of acne vulgaris. J Am Acad Dermatol. (2024) 90:1006.e1–1006.e30. doi: 10.1016/j.jaad.2023.12.017, 38300170

[4] Thiboutot D Gollnick H Bettoli V Dréno B Kang S Leyden JJ . New insights into the management of acne: an update from the global Alliance to improve outcomes in acne group. J Am Acad Dermatol. (2009) 60:S1–S50. doi: 10.1016/j.jaad.2009.01.019 19376456

[5] Del Rosso JQ Johnson SM Schlesinger T Green L Sanchez N Lain E . A randomized, controlled trial of Trifarotene plus doxycycline for severe acne vulgaris. J Clin Aesthet Dermatol. (2022) 15:E53–9. PMC9345187 35942016

[6] Barbieri JS Hoffstad O Margolis DJ. Duration of oral tetracycline-class antibiotic therapy and use of topical retinoids for the treatment of acne among general practitioners (GP): a retrospective cohort study. J Am Acad Dermatol. (2016) 75:1142–1150.e1. doi: 10.1016/j.jaad.2016.06.057, 27502311

[7] Straight CE Lee YH Liu G Kirby JS. Duration of oral antibiotic therapy for the treatment of adult acne: a retrospective analysis investigating adherence to guideline recommendations and opportunities for cost-savings. J Am Acad Dermatol. (2015) 72:822–7. doi: 10.1016/j.jaad.2015.01.048, 25752715

[8] Nast A Dréno B Bettoli V Degitz K Erdmann R Finlay AY . European evidence-based (S3) guidelines for the treatment of acne. J Eur Acad Dermatol Venereol. (2012) 26:1–29. doi: 10.1111/j.1468-3083.2011.04374.x, 22356611

[9] Begum D. Antibiotic resistance in acne treatment: emerging challenges and implications for dermatology practice in developing countries like Bangladesh. Sch Acad J Pharm. (2024) 13:377–85. doi: 10.36347/sajp.2024.v13i09.001

[10] Food and Drug Administration. Guidance on benzoyl peroxide; clindamycin phosphate topical gel (PSG_050756). (2023). Available online at: https://www.accessdata.fda.gov>drugsatfda_docs>psg (Accessed February 2023).

[11] Strauss JS Krowchuk DP Leyden JJ Lucky AW Shalita AR Siegfried EC . Guidelines of care for acne vulgaris management. J Am Acad Dermatol. (2007) 56:651–63. doi: 10.1016/j.jaad.2006.08.048 17276540

[12] Mahmud MR Akter S Tamanna SK Mazumder L Esti IZ Banerjee S . Impact of gut microbiome on skin health: gut-skin axis observed through the lenses of therapeutics and skin diseases. Gut Microbes. (2022) 14:2096995. doi: 10.1080/19490976.2022.2096995, 35866234 PMC9311318

[13] Jo JH Harkins CP Schwardt NH Portillo JA NISC Comparative Sequencing Program Zimmerman MD . Alterations of human skin microbiome and expansion of antimicrobial resistance after systemic antibiotics. Sci Transl Med. (2021) 13:eabd8077. doi: 10.1126/scitranslmed.abd8077 34936382 PMC8878148

[14] Swallow MA Fan R Cohen JM Bunick CG. Antibiotic resistance risk with Oral tetracycline treatment of acne vulgaris. Antibiotics (Basel). (2022) 11:1032. doi: 10.3390/antibiotics11081032, 36009899 PMC9405006

[15] Boyanova L Dimitrov G Raykova V Patrikov K Gergova R Markovska R. Cutibacterium acnes Phylotyping and antibiotic resistance to six antibiotics: a Bulgarian study. Microorganisms. (2025) 13:2185. doi: 10.3390/microorganisms13092185, 41011516 PMC12472480

[16] Aoki S Nakase K Hayashi N Nakaminami H Noguchi N. Increased prevalence of doxycycline low-susceptible Cutibacterium acnes isolated from acne patients in Japan caused by antimicrobial use and diversification of tetracycline resistance factors. J Dermatol. (2021) 48:1365–71. doi: 10.1111/1346-8138.15940, 33998707

[17] Sinnott SJ Bhate K Margolis DJ Langan SM. Antibiotics and acne: an emerging iceberg of antibiotic resistance? Br J Dermatol. (2016) 175:1127–8. doi: 10.1111/bjd.15129, 27996153

[18] Qiu W Liu T Liu X Chen H Luo S Chen Q . Enrofloxacin induces intestinal microbiota-mediated immunosuppression in zebrafish. Environ Sci Technol. (2022) 56:8428–37. doi: 10.1021/acs.est.1c08712, 35545936 PMC9228068

[19] Coenye T Spittaels KJ Achermann Y. The role of biofilm formation in the pathogenesis and antimicrobial susceptibility of *Cutibacterium acnes*. Biofilm. (2021) 4:100063. doi: 10.1016/j.bioflm.2021.100063, 34950868 PMC8671523

[20] Moura I Spittal W Clark E Ewin D Altringham J Fumero E . 224 profiling the effects of acne therapeutics, including the novel narrow-spectrum antibiotic sarecycline, on the human microbiota. J Invest Dermatol. (2021) 141:S187. doi: 10.1016/j.jid.2021.08.229 PMC9194605 35711781

[21] Chien AL Tsai J Leung S Mongodin EF Nelson AM Kang S . Association of systemic antibiotic treatment of acne with skin microbiota characteristics. JAMA Dermatol. (2019) 155:425–34. doi: 10.1001/jamadermatol.2018.5221, 30758497 PMC6459106

[22] Platt D Muller I Sufraz A Little P Santer M. Gps' perspectives on acne management in primary care: a qualitative interview study. Brit J Gen Pract. (2020) 71:e78–84. doi: 10.3399/bjgp20X713873, 33257464 PMC7716869

[23] Festok RA Ahuja AS Chen JY Chu L Barron J Case K . Barriers and facilitators affecting long-term antibiotic prescriptions for acne treatment. JAMA Dermatol. (2024) 160:535–43. doi: 10.1001/jamadermatol.2024.0203, 38568616 PMC10993164

[24] Patel DJ Bhatia N. Oral antibiotics for acne. Am J Clin Dermatol. (2021) 22:193–204. doi: 10.1007/s40257-020-00560-w, 32918267

[25] Whitehouse H Solman L Eady EA Layton AM. Oral antibiotics in acne: a retrospective single-centre analysis of current prescribing in primary care and its alignment with the national antibiotic quality premium. Br J Dermatol. (2019) 181:1341–2. doi: 10.1111/bjd.18339 31301232

[26] Stein Gold L Lain E Del Rosso JQ Gold M Draelos ZD Eichenfield LF . Clindamycin phosphate 1.2%/adapalene 0.15%/benzoyl peroxide 3.1% gel for moderate-to-severe acne: efficacy and safety results from two randomized phase 3 trials. J Am Acad Dermatol (2023); 89:927–935. doi: 10.1016/j.jaad.2022.08.069 37656094

[27] Margolis DJ Fanelli M Hoffstad O Lewis JD. Potential association between the oral tetracycline class of antimicrobials used to treat acne and inflammatory bowel disease. Am J Gastroenterol. (2010) 105:2610–6. doi: 10.1038/ajg.2010.303, 20700115

[28] Karimkhani Aksut C Schilling L Dellavalle R. Characterization of international oral antibiotic use for acne. J Invest Dermatol. (2019) 139:S43. doi: 10.1016/j.jid.2019.03.325

[29] Aslan Kayiran M Karadag AS Al-Khuzaei S Chen W Parish LC. Antibiotic resistance in acne: mechanisms, complications and management. Am J Clin Dermatol. (2020) 21:813–9. doi: 10.1007/s40257-020-00556-6, 32889707

