# Supplementary material for: Long-term impact of antibiotic exposure duration on recurrence and microbial resistance in moderate-to-severe acne: a real-world retrospective analysis
Source: Front Med (Lausanne). 2026 Mar 24;13:1782651. doi: 10.3389/fmed.2026.1782651 (PMC13079604; doi:10.3389/fmed.2026.1782651)
Supplement: Supplementary file 1 [file Table_1.DOCX]

**Supplementary Table 1** Severity-stratified acne recurrence at 12 months by antibiotic exposure duration

| **Recurrence Severity** | **Short-term**  **(n=80)** | **Standard-term**  **(n=80)** | **Prolonged**  **(n=80)** | **P for trend** |
| --- | --- | --- | --- | --- |
| No recurrence | 61 (76.3%) | 52 (65.0%) | 43 (53.8%) | <0.001 |
| Mild (IGA 2, 10-19 lesions) | 7 (8.8%) | 10 (12.5%) | 12 (15.0%) | 0.15 |
| Moderate (IGA 3, 20-49 lesions) | 10 (12.5%) | 14 (17.5%) | 19 (23.8%) | 0.003 |
| Severe (IGA 4, ≥50 lesions/nodules) | 2 (2.5%) | 4 (5.0%) | 6 (7.5%) | 0.09 |
| Any recurrence | 19 (23.8%) | 28 (35.0%) | 37 (46.3%) | <0.001 |

**Supplementary Table 2** Sensitivity analyses for the association between prolonged antibiotic exposure and 12-month recurrence

| **Analysis** | **Recurrence rate**  **(%) Short / Std / Prolonged** | **Adjusted HR**  **(95% CI)*** | **P-value** |
| --- | --- | --- | --- |
| Primary analysis (≥20 lesions) | 23.8 / 35.0 / 46.3 | 2.31 (1.31-4.07) | 0.004 |
| Alternative threshold: ≥30 lesions | 15.0 / 22.5 / 32.5 | 2.45 (1.28-4.68) | 0.007 |
| Alternative threshold: ≥10 lesions | 32.5 / 47.5 / 58.8 | 2.15 (1.25-3.70) | 0.006 |
| Baseline IGA ≥4 | 31.6 / 45.2 / 58.3 | 2.67 (1.34-5.31) | 0.005 |
| Excluding isotretinoin rescue | 22.7 / 33.8 / 44.4 | 2.19 (1.21-3.97) | 0.009 |
| Competing risk model† | — | 2.21 (1.26-3.87) | 0.005 |

HR = hazard ratio; all models compare prolonged vs. short-term exposure, adjusted for age, sex, baseline IGA, and cumulative dose (except IGA ≥4 analysis, which excludes baseline IGA from adjustment). †Subdistribution hazard ratio (sHR) from Fine-Gray model treating isotretinoin rescue as competing event.
